# Labiolingual thickness and cross‐sectional area in callitrichid incisors

**DOI:** 10.1111/joa.70162

**Published:** 2026-04-21

**Authors:** Keegan R. Selig

**Affiliations:** ^1^ Department of Anthropology Texas A&M University College Station Texas USA

**Keywords:** beam theory, bending resistance, *Callithrix*, dental signatures, exudativory

## Abstract

Several species of primate rely on tree exudates as a component of their diet, with taxa ranging from opportunistic exudativores to intensive exudativores. To access these exudates, many taxa make use of modified anterior teeth to gouge into tree bark to stimulate the flow of saps or gums. Recent efforts have identified several “dental signatures” of exudativory among primates, including differences in the robustness and bending resistance of the strepsirrhine toothcomb. The relative size and the labiolingual thickness of the lower incisors have also been cited as a difference between gouging and non‐gouging callitrichids, with the former predicted to have relatively larger (in cross‐sectional area) and labiolingually thicker lower incisors. However, along with methodological issues, these predictions have generally only been tested in a small sample of callitrichid taxa. Here, these predictions are tested in a sample of seven extant callitrichid species (*N* = 185 specimens), representing five genera. The results suggest that gouging callitrichids are not characterized by relatively larger incisors, contrary to previous results. However, the gougers are characterized by labiolingually thicker lower incisors compared to the non‐gougers, which hypothetically helps resist bending stress during gouging. Having relatively taller incisors, as well as more highly decussated enamel, larger tooth roots, and differences in enamel distribution, along with the labiolingually thicker lower incisors may explain why the gouging taxa are not characterized by relatively larger incisors. This pattern of incisor morphology serves as another signal for primate gouging and may provide insight into gouging behavior in the fossil record and other extant taxa such as the exudativorous lorisoids.

## Graphical abstract

Many primates gouge to extract gums or saps from trees as part of their diet. Gouging callitrichid monkeys are characterized by lower incisors that are labiolingually thicker than non‐gougers, however, their incisors are not any larger in cross‐section. This difference may provide key insight into the gouging behavior of fossil primates.

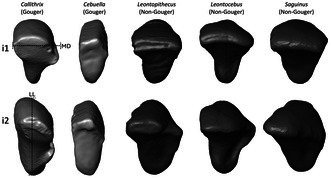

## 1 INTRODUCTION

Several primate species rely on tree exudates as a component of their diet (Génin et al., 2010; Nash, 1986; Rosenberger, 2010; Smith, 2010; Viguier, 2004). At least 69 species have been observed consuming exudates (Smith, 2010), with taxa ranging from opportunistic feeders (e.g., *Leontopithecus, Leontocebus, and Saguinus*), with exudates making up a small portion of the diet (1%‐ 16%), to intensive exudativores (e.g., *Callithrix* and *Cebuella*), where exudates make up the majority (>50%) of the diet (Nash, 1986; Smith, 2010). To access these exudates, many intensive exudativores actively gouge into trees to encourage the flow of saps and gums using modified front teeth (Burrows et al., 2015; Burrows & Nash, 2010; Burrows, Nash, Hartstone‐Rose, Selig, et al., 2020; Burrows, Nash, Hartstone‐Rose, Silcox, et al., 2020; López‐Torres et al., 2020; Rosenberger, 1978; Selig et al., 2019). It has been suggested that exudates may have been an important component of the diet in early primate species (Bearder & Martin, 1980; Burrows, Nash, Hartstone‐Rose, Selig, et al., 2020; López‐Torres et al., 2020; Martin, 1979; Selig et al., 2024). Therefore, there has been a large recent effort to identify dental signatures of exudativory to help answer questions surrounding the origin of this dietary behavior (Burrows et al., 2015; Burrows & Nash, 2010; Burrows, Nash, Hartstone‐Rose, Selig, et al., 2020; Burrows, Nash, Hartstone‐Rose, Silcox, et al., 2020; Selig et al., 2019, 2024).

Much of the work studying the form‐function relationship of the teeth and gouging has focused on exudativorous lorisoids, which are characterized by robust and bending‐resistant toothcombs, as well as uneven enamel distribution on the anterior teeth (Burrows et al., 2015; Burrows & Nash, 2010; Burrows, Nash, Hartstone‐Rose, Selig, et al., 2020; Burrows, Nash, Hartstone‐Rose, Silcox, et al., 2020; Selig et al., 2019). However, in most cases, it is difficult to distinguish lorisoids that actively gouge to extract gums (i.e., *Nycticebus*), and those that “scrape” bark or semi‐dried gum plugs to stimulate flow (i.e., *Euoticus* and *Otolemur*) (Selig et al., 2019). Although behavioral and morphological studies suggest that gouging callitrichids (marmosets) do not generate larger bite forces than non‐gouging tamarins but rather favor larger gapes (Vinyard et al., 2003, 2009; Vinyard & Ryan, 2006), their anterior teeth still likely experience different loading during gouging compared to non‐gouging callitrichids (Hogg et al., 2011). As such, marmosets are characterized by a suite of dental adaptations thought to be related to these gouging behaviors. For example, the marmosets *Callithrix* and *Cebuella* are characterized by tall lower incisors relative to the canines, whereas the non‐gouging tamarins have shorter incisors (Garber, 1992). This has led to the terms “short‐tusked” and “long‐tusked” being used to describe the tamarins and marmosets, respectively (Coimbra‐Filho & Mittermeier, 1977; Napier & Napier, 1967). Natori and Shigehara (1992) examined gouging and non‐gouging callitrichids and found that the gouging marmosets have larger incisors compared to the non‐gouging tamarins based on their measurement of incisor width compared to the area of the first and second molars. Deane and Agosto (2025) recently examined incisor size in a sample of frugivorous platyrrhines, concluding that larger incisors better resist stress; however, they did not include any callitrichids. Hogg et al. (2011) examined the absolute labiolingual thickness of the lower central incisors of *Callithrix jacchus* and *Leontocebus fuscicollis*. They found that *Callithrix* has absolutely thicker incisors, which they suggest helps the tooth function better as a wedge to propagate a crack in tree bark. In searching for dental signals of exudativory to study adaptations to this foraging strategy in fossil taxa, absolute incisor thickness likely does not provide much insight given that it is an absolute measurement. Like in the case of *Callithrix*, the anterior dentition of exudativorous strepsirrhines has generally been described as labiolingually thick (see Vinyard et al., 2003), and therefore potentially better able to resist bending compared to non‐exudativores (Burrows et al., 2015; Burrows & Nash, 2010; Burrows, Nash, Hartstone‐Rose, Silcox, et al., 2020). Deane (2015) examined incisor‐bending resistance in a sample of platyrrhines, and concluded that the frugivores, which theoretically experience higher stress to the anterior teeth when peeling fruits or nuts, have labiolingually thicker incisors. However, Deane (2015) did not include any callitrichids.

The following study is an examination of dental signatures of exudativorous gouging in the lower incisors of callitrichids. The following predictions are tested: (1) that gouging callitrichids are characterized by relatively larger incisor cross‐sectional areas. Theoretically, larger incisors mean better resisting the potential stresses of gouging the anterior teeth into a tree as the stresses are dissipated over a larger area (Deane & Agosto, 2025; Natori & Shigehara, 1992; Pollock et al., 2022). To date, there has been no taxonomically broad test of incisor size among callitrichids in the context of exudativory. (2) that gouging taxa will be characterized by lower incisors that are labiolingually thicker relative to their mesiodistal width compared to the non‐gougers. Following the principles of beam theory (Biknevicius et al., 1996), labiolingually thick incisors are hypothetically better at resisting labiolingual bending (Deane, 2015; Hogg et al., 2011; Pollock et al., 2022). For example, a 100% increase in labiolingual thickness (with mesiodistal length, crown height, and the force applied remaining consistent) should result in a 300% increase in bending resistance in the labiolingual direction, and a 100% increase in bending resistance in the mesiodistal direction (Deane, 2015). The theory suggests that adding more material in the plane of stress (labiolingually) helps reduce the stress experienced. Therefore, it is predicted that the gouging callitrichids will be characterized by labiolingually thick lower incisors to better resist the bending stresses experienced during gouging.

## 2 MATERIALS AND METHODS

**FIGURE 1:**
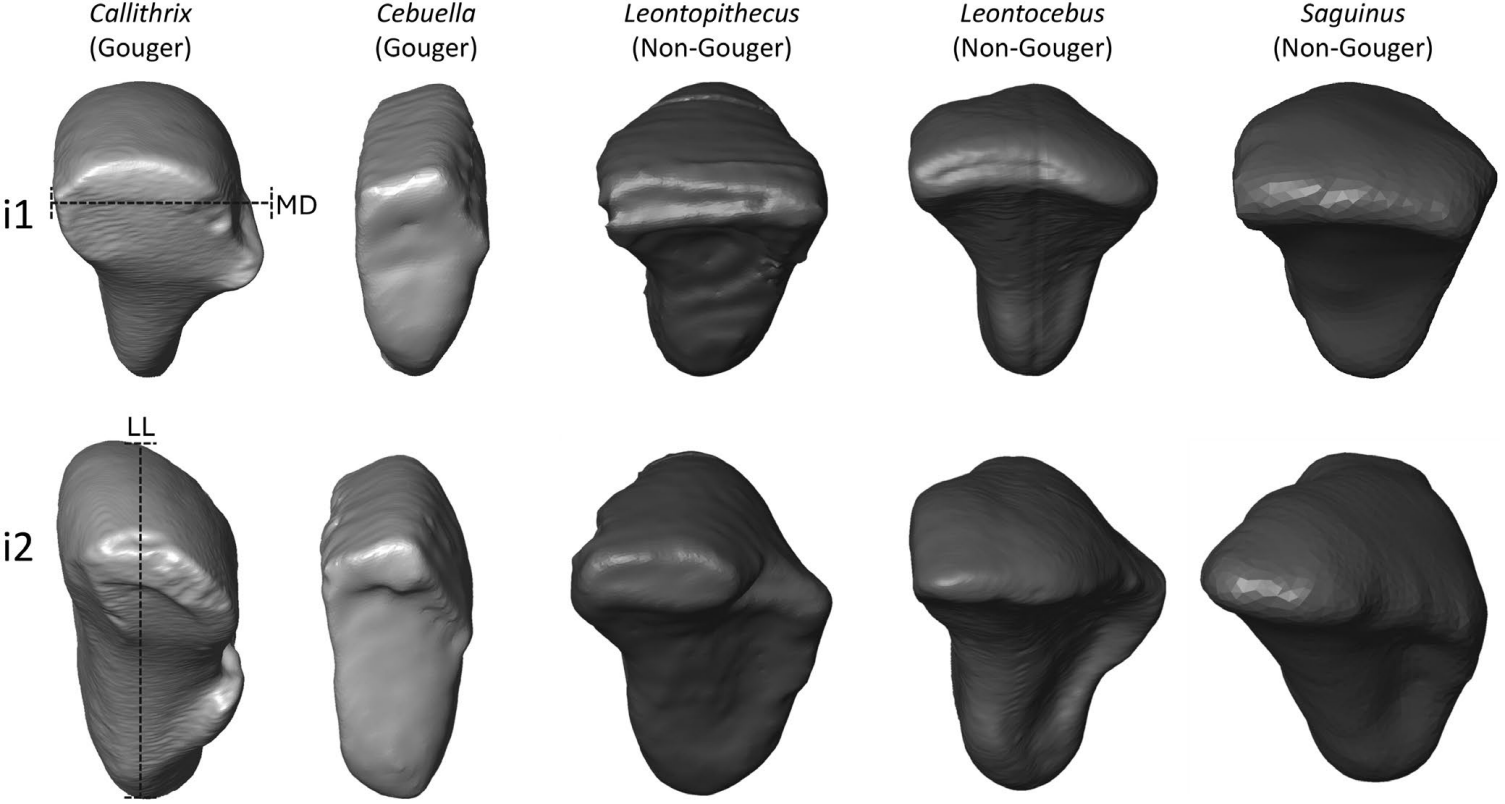
Representative three‐dimensional meshes of each genus included in the analysis. Meshes are *Callithrix jacchus* (AMNH 100249), *Cebuella pygmaea* (AMNH 76328), Leontopithecus rosalia (AMNH 235274), *Leontocebus fuscicollis* (AMNH 98304), and *Saguinus geoffroyi* (USNM 306848). Meshes are scaled to equal height. Meshes are from MorphoSource.org (Boyer et al., 2016). LL = labiolingual measurement, MD = mesiodistal measurement.

The sample (*N* = 185) includes measurements of the lower first (i1) and second (i2) incisors of 7 species of callitrichids (Figure 1) that vary in their degree of exudativory and in their exudate foraging behaviors (Table 1). Observational data suggest that marmosets (*Callithrix* and *Cebuella*) actively gouge to extract exudates, whereas tamarins (*Leontopithecus, Leontocebus*, and *Saguinus*) do not (Forsythe & Ford, 2011; Nash, 1986). Specimens were previously measured by Plavcan (1990) and were included here where a complete set of measurements (i.e., length and width) were available for the i1, the i2, and the m2 (Table S1). Area of the incisors was calculated as an oval, where the labiolingual length is divided by 2, multiplied by the mesiodistal width divided by 2, and multiplied by pi; the area of the molars was calculated as the buccolingual width (width at trigonid + width at talonid divided by 2) multiplied by the mesiodistal length. To examine potential bending resistance in the incisors, a third variable, the ratio of the labiolingual Length divided by the mesiodistal width was calculated (LL:MD). Following beam theory, taxa with higher LL:MD ratios are characterized by incisors that are labiolingually thicker than they are mesiodistally wide and are therefore theoretically better at resisting bending stresses (Deane, 2015).

**TABLE 1:** Taxa included in the analysis. Data on the exudate component of the diet come from Smith (2010).

Species	*N*	Exudate component	Gouger?
*Callithrix jacchus*	6	73.20%	Yes
*Callithrix penicillata*	7	70.00%	Yes
*Cebuella pygmaea*	35	67.00%	Yes
*Leontopithecus rosalia*	14	1.50%	No
*Leontocebus fuscicollis*	34	12.34%	No
*Saguinus geoffroyi*	36	14.40%	No
*Saguinu niger*	53	3.10%	No

All statistical analyses were performed in PAST 5.2.1 (Hammer et al., 2001). Ordinary least squares (OLS) regressions were used to examine the relationship between lower incisor area and m2 area. Differences between residuals were then examined at the species‐level using the Kruskal–Wallis and Bonferroni corrected Dunn's *post hoc* tests and between gougers and non‐gougers using the Mann–Whitney *U*. To test differences in LL:MD ratios between specimens grouped at the species‐level, the Kruskal–Wallis and Bonferroni corrected Dunn's *post hoc* tests were used, and to test differences between species grouped as gougers or non‐gougers, the Mann–Whitney *U* was used.

## 3 RESULTS

**FIGURE 2:**
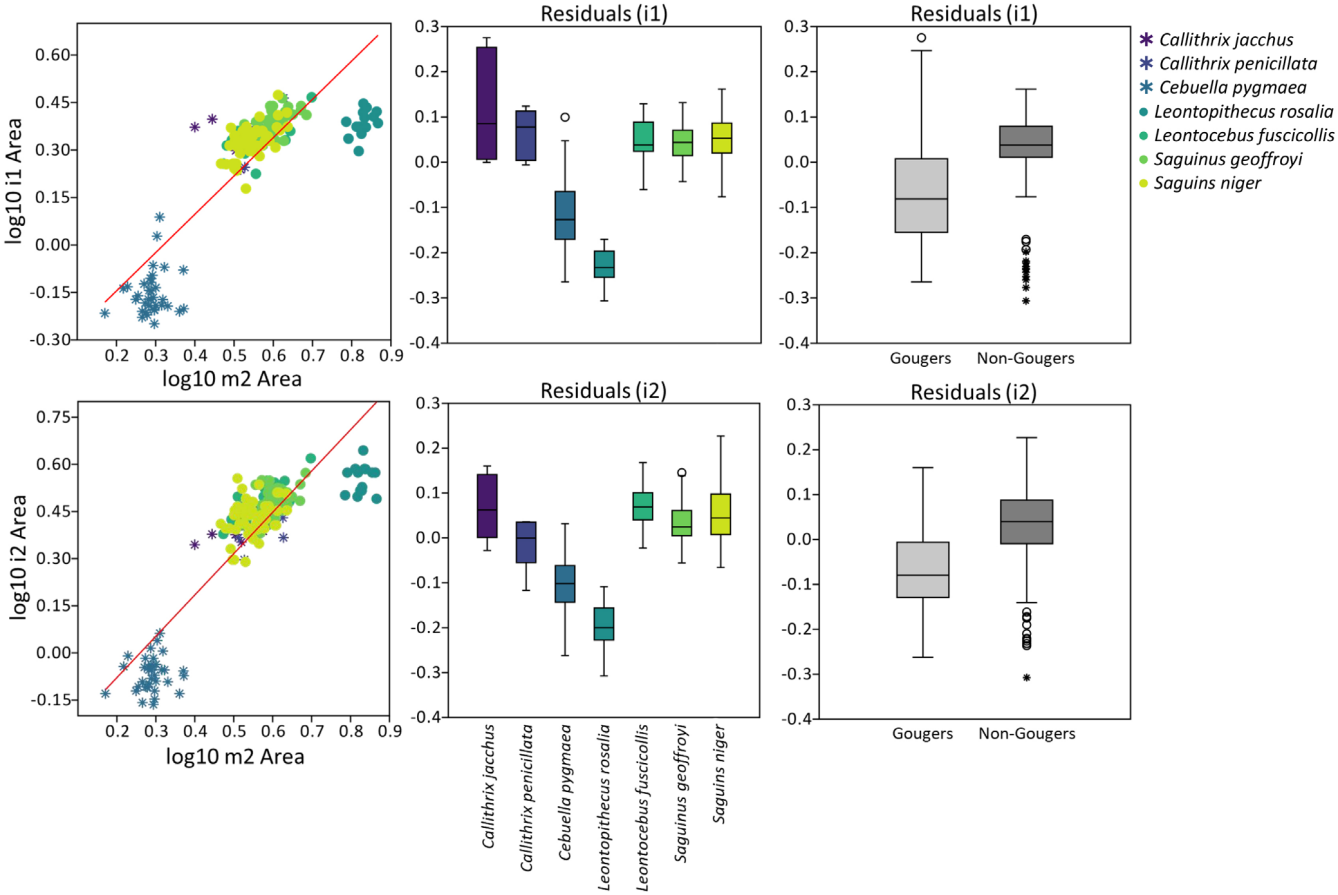
Top Row‐ Left: Plot of log10 m2 Area against log10 i1 Area with OLS regression line fitted. The stars represent gouging taxa; the filled circles represent non‐gouging taxa. Summary results of the OLS regression are presented in Table 3. Each point represents one specimen. Middle: Boxplots of the residuals for the OLS regression of log10 m2 Area against log10 i1 Area with specimens grouped by species. Right: Boxplots of the residuals for the OLS regression of log10 m2 Area against log10 i1 Area with specimens grouped as gougers or non‐gougers. Bottom Row‐ Left: Plot of log10 m2 Area against log10 i2 Area with OLS regression line fitted. The stars represent gouging taxa; the filled circles represent non‐gouging taxa. Summary results of the OLS regression are presented in Table 3. Each point represents one specimen. Middle: Boxplots of the residuals for the OLS regression of log10 m2 Area against log10 i2 Area with specimens grouped by species. Right: Boxplots of the residuals for the OLS regression of log10 m2 Area against log10 i2 Area with specimens grouped as gougers or non‐gougers. The whiskers are drawn from the top (and bottom) of the box up to the largest data point less than 1.5 times the box height or depth. Values outside this range are shown as circles, values further than 3 times above or below the box are shown as stars (Hammer et al., 2001).

The summary results of the calculation of lower first and second incisor area and molar area are presented in Table 2, as are the ratios of LL:MD for the incisors. The OLS regression of m2 and i1 area is presented in Figure 2 and Table 3. Although *Callithrix* has relatively large i1s, so too do the species of *Saguinus* and *Leontocebus*. Both the smallest species, *Cebuella*, and the largest species included, *Leontopithecus*, have small lower first incisors relative to m2 area. Comparison of residuals using the Kruskal–Wallis at the species level suggests that there are significant differences between species (*H* = 98.09, *p* = <0.001). The results of the Bonferroni corrected Dunn's *post hoc* test are presented in Table 4 and reflect the observation that *Cebuella* and *Leontopithecus* have significantly smaller central incisors. The Mann–Whitney *U* comparing residuals between gougers and non‐gougers suggests that the non‐gougers have significantly higher residuals (Gougers: *N* = 48, Mean Rank = 16.195, Non‐Gougers: *N* = 137, Mean Rank = 76.805, *p* = <0.001, z = 4.5965, *U* = 1820), and therefore relatively larger i1s compared to the gougers.

**TABLE 2:** Mean data and standard deviation (in brackets) for each species included in the analysis.

dSpecies	*N*	i1 LL:MD	i1 area	i2 LL:MD	i2 area	m2 area
*Callithrix jacchus*	6	1.515 (0.134)	2.139 (0.273)	1.836 (0.147)	2.370 (0.127)	3.141 (0.439)
*Callithrix penicillata*	7	1.375 (0.201)	2.332 (0.336)	1.844 (0.133)	2.407 (0.226)	3.676 (0.404)
*Cebuella pygmaea*	35	1.424 (0.254)	0.713 (0.133)	2.133 (0.228)	0.864 (0.109)	1.954 (0.174)
*Leontopithecus rosalia*	14	1.267 (0.087)	2.443 (0.222)	1.113 (0.147)	3.592 (0.375)	6.752 (0.366)
*Leontocebus fuscicollis*	34	1.192 (0.061)	2.185 (0.202)	1.040 (0.087)	2.895 (0.377)	3.645 (0.425)
*Saguinus geoffroyi*	36	1.185 (0.091)	2.472 (0.206)	1.035 (0.082)	3.095 (0.287)	4.049 (0.330)
*Saguinu niger*	53	1.227 (0.120)	2.136 (0.251)	1.024 (0.113)	2.696 (0.344)	3.530 (0.336)

Abbreviation: LL:MD = labiolingual:mesiodistal ratio.

**TABLE 3:** Results of the ordinary least squares regression of log–log transformed m2 area ~ incisor area.

OLA regression	m2 ~ i1	m2 ~ i2
Slope a	1.211	1.314
Std. error a	0.056	0.052
*P* (slope)	<0.001	<0.001
Intercept b	−0.387	−0.342
Std. error b	0.031	0.029
95% bootstrapped CI (*N* = 1999)		
Slope a	(1.036, 1.379)	(1.166, 1.461)
Intercept b	(−0.480, −0.300)	(−0.420, −0.264)
Correlation		
*R*	0.849	0.883
*R* ^2^	0.720	0.779
*T*	21.712	25.400
*P*	<0.001	<0.001
Permutation *P*	<0.001	<0.001

*Note*: Regression plotted in Figure 1.

**TABLE 4:** Bonferroni corrected Dunn's *post hoc* test on the OLS residuals of molar size and incisor size.

	*C. jacchus*	*C. penicillata*	*Ce. pygmaea*	*Lp. rosalia*	*L. fuscicollis*	*S. geoffroyi*	*S. niger*
*C. jacchus*		1.000	0.001	**<0.001**	1.000	1.000	1.000
*C. penicillata*	1.000		0.001	**<0.001**	1.000	1.000	1.000
*Ce. pygmaea*	**0.002**	1.000		1.000	**<0.001**	**<0.001**	**<0.001**
*Lp. rosalia*	**<0.001**	0.273	1.000		**<0.001**	**<0.001**	**<0.001**
*L. fuscicollis*	1.000	0.241	**<0.001**	**<0.001**		1.000	1.000
*S. geoffroyi*	1.000	1.000	**<0.001**	**<0.001**	1.000		1.000
*S. niger*	1.000	0.884	**<0.001**	**<0.001**	1.000	1.000	

*Note*: *p* values above the parallel are the i1 (see Figure 1) and below the parallel are the i2 (see Figure 2). Bold values are significant (*δ* = 0.05).

The OLS regression of m2 and i2 area is presented in Figure 2 and Table 3. The patterns observed for i1 are generally the same for the i2 with *Cebuella* and *Leontopithecus* characterized by smaller lower second incisors. Comparison of the residuals using the Kruskal–Wallis suggests that there is a significant difference between sample medians (*H* = 105, *p* = <0.001) at the species level. Results of the Bonferroni corrected Dunn's *post hoc* test are presented in Table 4. The observation that *Cebuella* and *Leontopithecus* have smaller lateral incisors is supported by these results. The Mann–Whitney *U* testing for differences in residuals between the gougers and non‐gougers suggests that the non‐gougers have significantly higher residuals (Gougers: *N* = 48, Mean Rank = 13.681, Non‐Gougers: *N* = 137, Mean Rank = 79.319, *p* = <0.001, z = 6.053, *U* = 1355), and therefore relatively larger i2s compared to the gougers.

**FIGURE 3:**
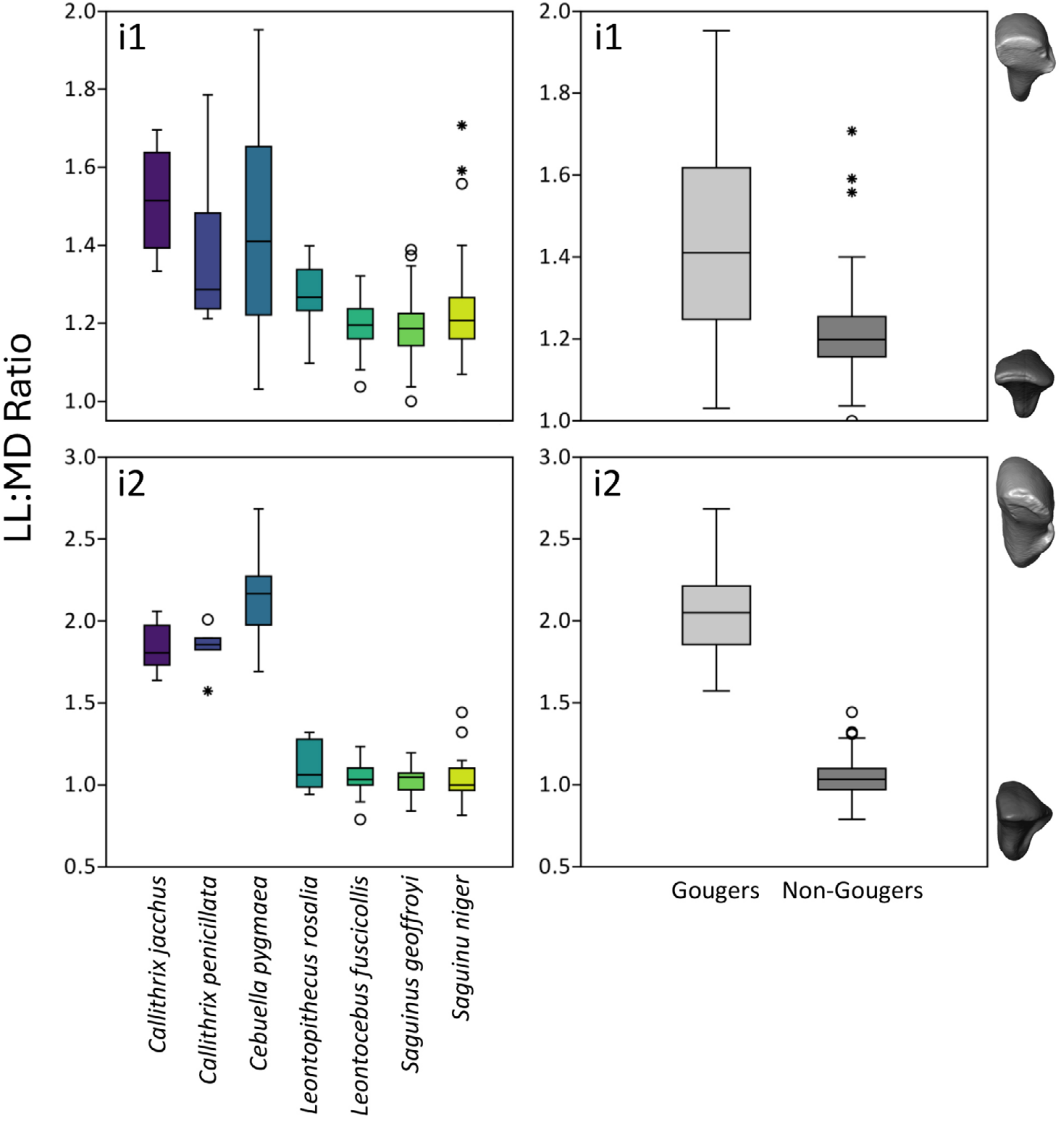
Box plots of labiolingual width divided by mesiodistal length (LL:MD ratio) for the i1 (top row) and the i2 (bottom row) with specimens grouped by species (left column) and as gougers or non‐gougers (right column). The representative tooth meshes are reconstructed micro‐CT scans of *Callithrix jacchus* (AMNH 100249) in light gray (i1 in the top row, i2 in the bottom row) and of *Leontocebus fuscicollis* (AMNH 98304) in dark gray (i1 in the top row, i2 in the bottom row), which were originally scanned and analyzed in Burrows, Nash, Hartstone‐Rose, Silcox, et al. (2020). Meshes are scaled to equal widths.

The LL:MD ratios are plotted at the species‐level and among gougers and non‐gougers in Figure 3. At the species level, both species of *Callithrix* as well as *Cebuella* generally have higher LL:MD ratios for the i1 and the i2. The Kruskal–Wallis comparing the LL:MD ratios at the species‐level for the i1 suggests that there is a significant difference between sample medians (*H* = 50.63, *p* = <0.001). Results of the Bonferroni corrected Dunn's *post hoc* test are presented in Table 5. *Callithrix jacchus* differs significantly from all non‐gougers, with the exception of *Leontopithecus*, whereas *C. penicillata* differs from only *S. geoffroyi*. *Cebuella* is also significantly different in its i1 LL:MD ratio from all non‐gouging taxa, with the exception of *Leontopithecus*. The Kruskal–Wallis comparing the LL:MD ratios at the species‐level for the i2 suggests that there is a significant difference between sample medians (*H* = 109.1, *p* = <0.001). Results of the Bonferroni corrected Dunn's *post hoc* test are presented in Table 5 and suggest that the observed higher LL:MD ratios in both species of *Callithrix* differ from all non‐gougers, with the exception of *Leontopithecus*, whereas *Cebuella* differs significantly from all non‐gougers. The Mann–Whitney *U* comparing LL:MD in gougers and non‐gougers suggests that the gougers have significantly higher LL:MD of the i1 (Gougers: *N* = 48, Mean Rank = 35.049, Non‐Gougers: *N* = 137, Mean Rank = 57.951, *p* = <0.001, z = 6.328, *U* = 1268), and of the i2 (Gougers: *N* = 48, Mean Rank = 41.903, Non‐Gougers: *N* = 137, Mean Rank = 51.097, *p* = <0.001, z = 10.311, *U* = 0), compared to the non‐gougers.

**TABLE 5:** Bonferroni corrected Dunn's *post hoc* test on of differences between species in their LL:MD ratios.

	C. Jacchus	C. Penicillata	Ce. Pygmaea	Lp. Rosalia	L. fuscicollis	S. Geoffroyi	S. Niger
C. jacchus		1.000	1.000	0.879	**0.002**	**0.001**	**0.008**
C. penicillata	1.000		1.000	1.000	0.071	**0.032**	0.295
Ce. pygmaea	1.000	1.000		1.000	**<0.001**	**<0.001**	**0.001**
Lp. rosalia	0.327	0.201	**<0.001**		0.346	0.140	1.000
L. fuscicollis	**0.026**	**0.010**	**<0.001**	1.000		1.000	1.000
S. geoffroyi	**0.017**	**0.006**	**<0.001**	1.000	1.000		1.000
S. niger	**0.004**	**0.001**	**<0.001**	1.000	1.000	1.000	

*Note*: Bold values are significant (*δ* = 0.05). *p* values above the parallel are for the i1 and below the parallel are the i2.

## 4 DISCUSSION

The results of the current study do not support the first prediction: that gouging callitrichids are characterized by relatively larger incisors. Previous works suggested that gouging taxa have larger incisors (Natori & Shigehara, 1992) and analysis of frugivorous platyrrhines supports this idea (Deane & Agosto, 2025). Natori and Shigehara (1992) examined incisor size as the width of the incisors relative to the width of the first molar + the width of the second molar. Molar width is significantly genetically correlated with factors such as body size and is therefore more likely to be related to growth and nutrition compared to molar length (Hlusko et al., 2006). Perhaps only considering width and using a combination of two molars as the denominator in their relative measurement of size provided different results than those of the present study. While it was predicted that larger incisors would better reduce the stress of a given force and would be associated with gouging (if gouging does indeed result in high stress), it is possible that other adaptations provide the necessary buffer. For example, more highly decussated enamel observed in marmosets (Hogg et al., 2011) may hypothetically help strengthen the enamel and reduce the risk of tooth fracture. Additionally, larger and more surface area of the anterior tooth roots relative to symphyseal bone in marmosets (Hogg et al., 2011) may help dissipate the stresses of gouging. Finally, differences in enamel distribution observed on the anterior teeth of gougers (thick labial enamel, think or absent lingual enamel, Rosenberger, 1978; Selig et al., 2019) are hypothesized to provide a chisel‐like edge to the teeth that help propagate a crack in the tree (Rosenberger, 1978; Selig et al., 2019) and to reduce the maximum principal stress on the lingual aspect of the tooth during bending (Kupczik & Chattah, 2014).

Whereas the first prediction was not supported, the results of the current study generally support the second prediction: that gouging taxa are characterized by lower incisors that are labiolingually thicker relative to their mesiodistal width compared to the non‐gougers. Hogg et al. (2011) suggest that the gouging *Callithrix* has absolutely thicker incisors compared to the non‐gouging *Leontocebus*. Following the predictions and principles of beam theory (Biknevicius et al., 1996), labiolingually thick incisors are hypothetically better at resisting bending (Deane, 2015; Hogg et al., 2011), which is likely an important adaptation if the lower teeth are wedged into trees to propagate a crack. Given that incisor width was calculated here as a relative measure (labiolingual width divided by mesiodistal length), this metric serves as a useful dental signature for reconstructing gouging behavior in the fossil record of callitrichids, which is particularly useful given the fragmentary nature of the fossil record (Rosenberger et al., 1990; Setoguchi & Rosenberger, 1985). This method may also serve as a useful signature of gouging in other taxa such as the lorisoids. Although Burrows et al. (2015); Burrows, Nash, Hartstone‐Rose, Silcox, et al. (2020) and Burrows and Nash (2010) suggest that gouging lorisoids do have higher bending resistance in the toothcomb, their method relies on measurement of the entire toothcomb. Extinct taxa such as *Karanisia* are not known to preserve a complete toothcomb (López‐Torres et al., 2020), therefore, a method that is intrinsic to a single tooth may provide additional insight into potential gouging behavior in extinct taxa as well. Finally, this method may be useful for reconstructing other gouging behaviors in the fossil record, such as insect extracting, like in *Daubentonia*, or tree‐gouging for scent marking, like in *Propithecus* (Miaretsoa et al., 2023). In the broader context of primate evolution, however, many primate taxa are characterized by labiolingually thick lower incisors (Deane & Agosto, 2025), largely as an adaptation for peeling fruit and seeds. Therefore, study of labiolingual incisor thickness may be most useful in the context of exudativory in clades such as the callitrichids, and potentially lorisoids (Vinyard et al., 2003).

## Supporting information


**Table S1.** Raw data used in the analysis. Data are originally from Plavcan (1990).

## Data Availability

All data considered in this analysis were originally collected by Plavcan (1990) and are presented in Table S1.
